# Posterior fossa and spinal gangliogliomas form two distinct clinicopathologic and molecular subgroups

**DOI:** 10.1186/2051-5960-2-18

**Published:** 2014-02-14

**Authors:** Kirti Gupta, Wilda Orisme, Julie H Harreld, Ibrahim Qaddoumi, James D Dalton, Chandanamali Punchihewa, Racquel Collins-Underwood, Thomas Robertson, Ruth G Tatevossian, David W Ellison

**Affiliations:** 1 Department of Pathology, St. Jude Children’s Research Hospital, 262 Danny Thomas Place, Memphis, TN 38105, USA; 2 Department of Radiological Sciences, St. Jude Children’s Research Hospital, 262 Danny Thomas Place, Memphis, TN 38105, USA; 3 Department of Oncology, St. Jude Children’s Research Hospital, 262 Danny Thomas Place, Memphis, TN 38105, USA; 4 Department of Pathology, Royal Brisbane and Women’s Hospital, Butterfield Street, Herston, QLD 4029, Australia

**Keywords:** Ganglioglioma, Pilocytic astrocytoma, Glioneuronal, BRAF, Mutation

## Abstract

**Background:**

Gangliogliomas are low-grade glioneuronal tumors of the central nervous system and the commonest cause of chronic intractable epilepsy. Most gangliogliomas (>70%) arise in the temporal lobe, and infratentorial tumors account for less than 10%. Posterior fossa gangliogliomas can have the features of a classic supratentorial tumor or a pilocytic astrocytoma with focal gangliocytic differentiation, and this observation led to the hypothesis tested in this study - gangliogliomas of the posterior fossa and spinal cord consist of two morphologic types that can be distinguished by specific genetic alterations.

**Results:**

Histological review of 27 pediatric gangliogliomas from the posterior fossa and spinal cord indicated that they could be readily placed into two groups: classic gangliogliomas (group I; n = 16) and tumors that appeared largely as a pilocytic astrocytoma, but with foci of gangliocytic differentiation (group II; n = 11). Detailed radiological review, which was blind to morphologic assignment, identified a triad of features, hemorrhage, midline location, and the presence of cysts or necrosis, that distinguished the two morphological groups with a sensitivity of 91% and specificity of 100%. Molecular genetic analysis revealed *BRAF* duplication and a *KIAA1549-BRAF* fusion gene in 82% of group II tumors, but in none of the group I tumors, and a BRAF:p.V600E mutation in 43% of group I tumors, but in none of the group II tumors.

**Conclusions:**

Our study provides support for a classification that would divide infratentorial gangliogliomas into two categories, (classic) gangliogliomas and pilocytic astrocytomas with gangliocytic differentiation, which have distinct morphological, radiological, and molecular characteristics.

## Background

Gangliogliomas are rare mixed glioneuronal tumors, composed of neoplastic glial and neuronal cells and representing 0.5-1.7% of all neuroepithelial tumors in the central nervous system (CNS) [1-4]. However, they constitute up to 4% of CNS tumors in the pediatric population and are the commonest tumor associated with chronic intractable focal epilepsy. Gangliogliomas are found throughout the CNS, but most (>70%) are localized to the temporal lobe, while they are uncommon in the posterior fossa (~5%) and spinal cord (~3%) [2,5-9]. Gangliogliomas in the cerebral lobes are often circumscribed tumors and amenable to complete surgical resection, which is reflected in good survival data [10]. Gangliogliomas in the posterior fossa or spinal cord have a poorer outcome, but it is unclear whether anatomic location or an inherent variance in biologic behavior accounts for this difference [1,6,10].

Genetic alterations in elements of the mitogen-activated protein kinase (MAPK) signaling pathway have been identified in many low-grade neuroepithelial tumors, including pilocytic astrocytoma (PA), pleomorphic xanthoastrocytoma (PXA), and ganglioglioma [11-14]. Recent studies have demonstrated that specific mutations are enriched in certain tumors; for example, *KIAA1549-BRAF* fusions are found in PAs, and BRAF:p.V600E mutations are frequently detected in PXAs (~70%) [14-19]. BRAF:p.V600E mutations are also present in about one quarter of gangliogliomas [14].

Through the neuropathology referral practice at St. Jude Children’s Research Hospital, we occasionally review the pathology of infratentorial gangliogliomas that demonstrate the features of a classic pilocytic astrocytoma, except for one or two collections of dysmorphic ganglion cells that are clearly part of the neoplastic process. This observation led to the hypothesis tested in this study; gangliogliomas of the posterior fossa and spinal cord consist of two morphologic types that can be distinguished by their molecular genetic alterations.

## Methods

The study cohort consisted of 27 WHO grade I gangliogliomas arising in the posterior fossa or spinal cord. Clinical and radiological features were compiled (Table 1). Median age at diagnosis was 10 years (range: 0.6 - 21 years), and the female:male ratio was 14:13. No patient fulfilled clinical criteria for the diagnosis of NF-1. Review of magnetic resonance imaging (MRI) was undertaken by one radiologist, who was blinded to pathology review and morphologic group assignment. Tumors were evaluated radiologically on the following parameters: location (dominant and secondary sites of involvement), relationship to midline, circumscription, extent of edema and restricted diffusion, and the presence of cysts or necrosis, hemorrhage, and enhancement. The study was conducted with St. Jude Children's Research Hospital Institutional Review Board approval (XPD07-107).

**Table 1:** Clinical and radiological data for two morphological groups of ganglioglioma

	**Pathology group**	**Age @ diagNosis (years)**	**Gender**	**Dominant site of tumor**	***BRAF*: p.V600E**	***BRAF *duplication**	***KIAA1549-BRAF fusion gene***	**Neuroimaging**						
								**Midline location**	**Circumscribed**	**Cysts/necrosis**	**Hemorrhage**	**Enhancement**	**Edema**	**Restricted diffusion**
GG01	I	6	M	Cerebellar hemisphere	Yes	No	No	No	No	0	0	+	+	No
GG02	I	9	F	Cerebellum	Yes	No	No	n/a						
GG03	I	21	F	Cerebellar hemisphere	Yes	No	No	No	Yes	0	0	++	+++	Yes
GG04	I	9	F	Medulla	Yes	No	n/a	No	No	0	0	+++	0	No
GG05	I	8	F	Medulla	Yes	No	n/a	No	Yes	0	0	++	0	n/a
GG06	I	8	M	Medulla	Yes	No	n/a	No	No	0	0	+++	+	Yes
GG07	I	15	F	MCP	Yes	No	No	No	No	0	0	+++	++	No
GG08	I	8	F	Cerebellar hemisphere	No	No	No	No	No	+++	0	+	0	No
GG09	I	11	F	MCP	No	No	n/a	No	Yes	0	0	++++	0	n/a
GG10	I	11	F	Medulla	No	No	n/a	No	No	0	0	++	++	n/a
GG11	I	12	M	Medulla	No	No	No	No	No	0	0	+++	++	Yes
GG12	I	1.8	M	Pons	No	No	No	No	No	0	0	+	++	No
GG13	I	21	M	MCP	No	No	No	No	No	+	0	+++	+	n/a
GG14	I	0.6	M	MCP	No	No	No	No	No	0	0	+	+	No
GG15	I	15	F	Cervico-medullary	No	No	No	n/a						
GG16	I	14	M	Medulla	No	No	No	No	No	0	0	++++	++	No
GG17	II	12	M	Vermis	No	Yes	Yes - ex16:ex9	No	Yes	++	0	+	0	Yes
GG18	II	4	F	Vermis	No	Yes	Yes - ex15:ex9	Yes	Yes	+	+	+++	+	Yes
GG19	II	12	F	Cord (thoraco-lumbar)	No	Yes	Yes - ex15:ex9	Yes	No	+	+	++	0	n/a
GG20	II	16	F	Cord (cervico-thoracic)	No	Yes	Yes - ex15:ex9	Yes	Yes	+++	0	++	+++	n/a
GG21	II	18	M	Cord (cervico-thoracic)	No	Yes	Yes - ex15:ex9	Yes	No	+++	+	+	++++	n/a
GG22	II	9	F	Vermis	No	Yes	Yes - ex15:ex9	No	Yes	++	0	+++	++	No
GG23	II	17	F	Vermis	No	Yes	Yes - ex16:ex9	No	Yes	++	+	+	++	Yes
GG24	II	10	M	Cord (cervical)	No	Yes	Yes - ex16:ex11	Yes	Yes	+++	+	++	++	n/a
GG25	II	9	M	Vermis	No	Yes	Yes - ex16:ex11	Yes	No	++++	0	++++	+	Yes
GG26	II	4	M	Medulla	No	No	No	No	No	0	0	+++	++	Yes
GG27	II	9	M	Midbrain	No	No	No	No	Yes	+++	0	+	++	n/a

M = male; F = female.

MCP = middle cerebellar peduncle.

n/a = Not available.

0 - ++++; magnitude scale.

### Histology and immunohistochemistry

Standard histological preparations, 4 μm formalin-fixed paraffin-embedded (FFPE) sections stained with hematoxylin & eosin were supplemented with immunohistochemical preparations. Antibodies to the following proteins were utilized for routine pathologic evaluation: glial fibrillary acidic protein (1:400, Dako M076101), synaptophysin (1:400, Leica MCL-L-SYNAP-299), NEU-N (1:5000, Chemicon MAB377), neurofilament protein (1:100, Dako M076229), microtubule-associated protein 2 (MAP2 1:10,000, Sigma M4403), and Ki67 (1:200, Dako M7240).

### Interphase fluorescence in situ hybridization (iFISH)

Dual-color iFISH was performed on 4 μm FFPE tissue sections. Probes were derived from BAC clones (BACPAC Resources, Oakland, CA), labeled with an AlexaFluor-488 or AlexaFluor-555 fluorochrome, and validated on normal control metaphase spreads to confirm chromosomal location. BAC clones RP11-96I22 and RP11-837G3 were used to screen for *BRAF* duplication at 7q34 (control probe on 7p11, RP11-251I15 and RP11-746C13). RP11-837G3 and RP11-948O19 were used in a ‘break-apart’ probe strategy to screen for *BRAF* rearrangement. RP11-265 F21 and RP11-297 N18 (*ETV6*), along with RP11-96B23 and RP11-948I15 (*NTRK3*), were used to screen for *ETV6-NTRK3* fusions.

### Nucleic acid extraction and mutation analysis

Genomic DNA was extracted from 10 μm FFPE scrolls, using the Maxwell® 16 Plus LEV DNA purification kit (Promega, Madison WI), and total RNA was extracted from FFPE scrolls using the Maxwell® 16 RNA FFPE prototype extraction kit (Promega, Madison WI), according to manufacturer’s instructions. BRAF:p.V600, KRAS:p.G12, and KRAS:p.Q61 were sequenced in genomic DNA using previously published primers [12]. PCRs were performed using GoTaq® Long PCR Master Mix (Promega, Madison, WI). All PCR products were visualized using 1% agarose gels. Direct sequencing of PCR products was performed using BigDye version 3.1 and a 3730XL DNA analyzer (Applied Biosystems, Foster City, CA). Results were screened using CLC Main Workbench sequence analysis software version 6.0.2 (CLC bio, Cambridge, MA).

### Real-time quantitative reverse-transcription PCR (qRT-PCR) for *KIAA1549-BRAF* detection

First-strand cDNA was synthesized using 1 μg total RNA in a 20 μL reaction mixture using the iScript cDNA synthesis kit (Bio-Rad Laboratories, Hercules, CA). The mixture was incubated at 25°C for 5 min, 42°C for 30 min, and 85°C for 5 min. qRT-PCR was performed using TaqMan reagents and the Applied Biosystems 7500 Real-Time PCR system (Life Technologies, Carlsbad, CA). Forward and reverse primers and TaqMan probes are listed in Table 2. *KIAA1549-BRAF* probes were labeled with 6-carboxyfluorescein (6-FAM) as a 5’ reporter dye and 6-carboxytetramethylrhodamine (TAMRA) as the 3′ quencher dye. A 10 μL aliquot of cDNA (corresponding to 100 ng of total RNA) was added to the PCR reaction mix to reach a final volume of 50 μL containing 25 μL of TaqMan Fast Universal PCR Master Mix (2X) (Roche Diagnostics, Indianapolis, IN), 300 nM of each forward and reverse primer, and 50 nM of TaqMan probe. Human GAPDH (Life Technologies, Carlsbad, CA) was used as an internal control. The thermal cycling conditions were 2 min at 50°C, 10 min at 95°C for denaturation, and 50 cycles at 95°C for 15 s followed by 60°C for 60s for annealing and extension. Presence of the fusion product was indicated by the appearance of signal above the critical threshold (Ct). All experiments were performed in duplicate.

**Table 2:** **Primers and TaqMan probes for *KIAA1549-BRAF *** **fusion gene variants**

**Gene fusion**	**Primer**	**TaqMan probe**
*KIAA1549-BRAF (KIAA exon 13 - BRAF exon 11) forward*	GGGTCCCCAGTAAGATCCAG	ATCGCCATGCAGCCGATCCCGGCACCT
*KIAA1549-BRAF (KIAA exon 13 - BRAF exon 11) reverse*	CTCGAGTCCCGTCTACCAAG	
*KIAA1549-BRAF (KIAA exon 15 - BRAF exon 9) forward*	CGTCCACAACTCAGCCTACATC	ACCACAGGTTTGTCTGC
*KIAA1549-BRAF (KIAA exon 15 - BRAF exon 9) reverse*	CCTGGAGATTTCTGTAAGGCTTTC	
*KIAA1549-BRAF (KIAA exon 15 - BRAF exon 11) forward*	AGCGATGGCACCTACAGGA	CGTCCACAACTCAGCCTACATCGGATGCCCA
*KIAA1549-BRAF (KIAA exon 15 - BRAF exon 11) reverse*	TCATCACTCGAGTCCCGTCT	
*KIAA1549-BRAF (KIAA exon 16 - BRAF exon 9) forward*	CCAGACGGCCAACAATCC	ACCACAGGTTTGTCTGC
*KIAA1549-BRAF (KIAA exon 16 - BRAF exon 9) reverse*	CCTGGAGATTTCTGTAAGGCTTTC	
*KIAA1549-BRAF (KIAA exon 16 - BRAF exon 10) forward*	CAGTGGGGGTCCTTCTACAG	AGCCCAGACGGCCAACAATCCCTGCAG
*KIAA1549-BRAF (KIAA exon 16 - BRAF exon 10) reverse*	CTTCCTTTCTCGCTGAGGTC	
*KIAA1549-BRAF (KIAA exon 16 - BRAF exon 11) forward*	AGTGGGGGTCCTTCTACAGC	AGCCCAGACGGCCAACAATCCCTGCAG
*KIAA1549-BRAF (KIAA exon 16 - BRAF exon 11) reverse*	CATGCCACTTTCCCTTGTAG	
*KIAA1549-BRAF (KIAA exon 17 - BRAF exon 10) forward*	GAATGACTCCCCCGACG	ACCACAGGTTTGTCTGCTACCCCCCCTGC
*KIAA1549-BRAF (KIAA exon 17 - BRAF exon 10) reverse*	AGGCTTTCACGTTAGTTAGTGAGC	
*KIAA1549-BRAF (KIAA exon 18 - BRAF exon 10) forward*	TGCTGCCAGAGGGATCTACTC	ACCACAGGTTTGTCTGC
*KIAA1549-BRAF (KIAA exon 18 - BRAF exon 10) reverse*	CCTGGAGATTTCTGTAAGGCTTTC	
*KIAA1549-BRAF (KIAA exon 19 - BRAF exon 9) forward*	CCAGGCTGGCCTTCGTAC	ACCACAGGTTTGTCTGC
*KIAA1549-BRAF (KIAA exon 19 - BRAF exon 9) reverse*	CCTGGAGATTTCTGTAAGGCTTTC	

## Results

### Histopathological features

Evaluation of histopathology and group assignment took place before the results of molecular analyses were established. Even though all tumors (n = 27) contained a low-grade glial element and a population of dysmorphic ganglion cells and thus qualified for a diagnosis of ganglioglioma, they could be readily assigned to two groups on the basis of their histopathological features.

#### Group I - classic ganglioglioma

**Figure 1:**
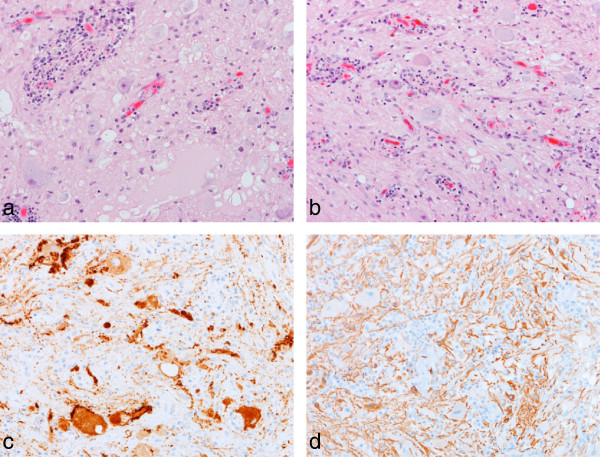
**Group 1 tumors – classic ganglioglioma.** The classic pathologic features of a ganglioglioma are demonstrated **(a, b)**, including perivascular aggregates of lymphoid cells, dysmorphic ganglion cells, and a fibrillary glial cell component. Immunoreactivity for synaptophysin highlights ganglion cells and their abnormal neuritic processes **(c)**, while the glial component is GFAP-positive **(d)**. All images, x200.

Tumors belonging to group I (16/27; 59%) contained dysmorphic ganglion cells and atypical glial cells, which were admixed throughout most of the tumor (Figure 1). Many group I tumors (13/16; 81%) exhibited aggregates of perivascular lymphocytes. Eosinophilic granular bodies were present in 7 of 16 tumors, but Rosenthal fibers were present in only three tumors. The supporting matrix varied from a reticulin-rich fibrous network, occasionally forming a lobular configuration, to a fine fibrillary component with variable cystic degeneration. The pleomorphism shown by neoplastic ganglion cells in group I tumors appeared greater than that of ganglion cells in group II tumors. Multinucleation in ganglion cells was a feature of several tumors in this group. The glial element in group I tumors was varied; it showed a fibrillary phenotype in most cases (10/16), but an admixed fibrillary and pilocytic phenotype in remaining cases. The fibrillary component diffusely infiltrated adjacent parenchyma in several tumors. Anaplastic features, including significant mitotic activity, were not detected, and there was no necrosis.

#### Group II - pilocytic astrocytoma with focal gangliocytic differentiation

**Figure 2:**
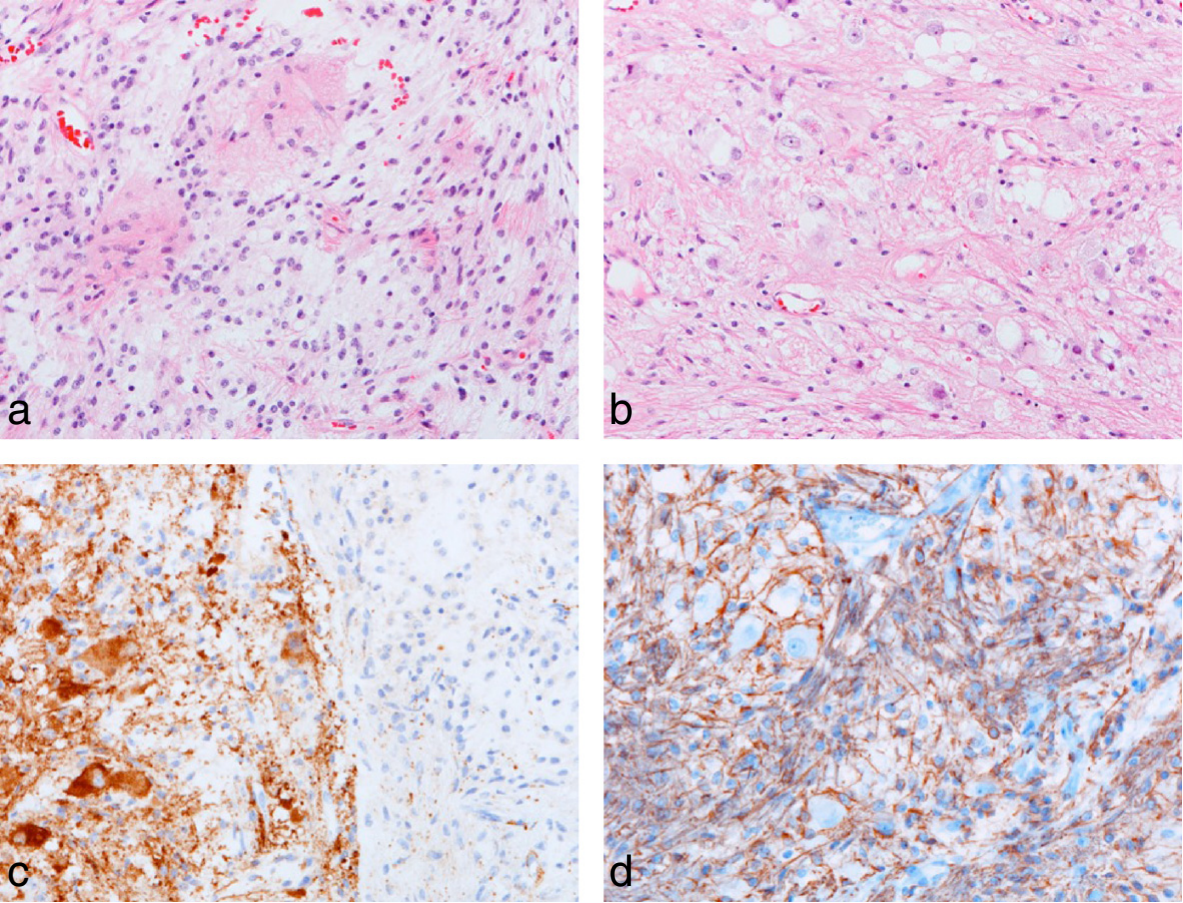
**Group II tumors - pilocytic astrocytoma with focal gangliocytic differentiation.** The classic pathologic features of a posterior fossa pilocytic astrocytoma **(a)** combines focally with collections of dysmorphic ganglion cells **(b)**. The edge of a gangliocytic nodule is highlighted by immunoreactivity for synaptophysin **(c)**. An admixed GFAP-positive pilocytic and fibrillary astrocytic component surrounds a few dysmorphic ganglion cells **(d)**. All images, x200.

Tumors in group II (n = 11/27; 41%) were largely characterized by the features of a classic pilocytic astrocytoma, but all had foci of gangliocytic differentiation (Figure 2). All tumors displayed a glial element with a biphasic architecture, which alternated between solid areas composed of piloid cells and cystic regions showing variable myxoid degeneration and containing disaggregated cells with a piloid or astrocytic phenotype. Four tumors contained a few areas where neoplastic glial cells showed an oligodendroglial phenotype. Variable numbers of Rosenthal fibers were found in the majority of tumors. Gangliocytic differentiation manifested as distinct clusters of haphazardly arranged dysmorphic ganglion cells in just one or two regions of the tumor. These cells were atypical and clearly part of the neoplastic process, occurring in areas that did not incorporate adjacent parenchyma. Bi-nucleation was a feature of ganglion cells in two tumors. Microvascular proliferation of the type seen in pilocytic astrocytomas was detected in several tumors, and two contained small foci of necrosis. The was no rosette formation.

Immunohistochemistry gave the expected results across both groups of tumors. Many neoplastic glial cells were GFAP-positive, while ganglion cells showed immunoreactivities for MAP2, synaptophysin and neurofilament proteins (Figures 1 and 2). NEU-N was expressed weakly by a few ganglion cells in group I tumors, and to a variable extent in ganglion cells in group II tumors. Ki67 immunolabeling was low in all tumors.

### Molecular features

**Figure 3:**
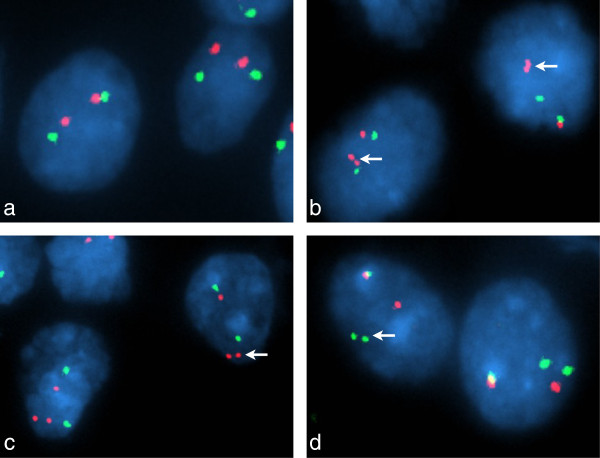
**Interphase fluorescence *in situ *hybridization analysis of the *BRAF *locus.** FISH probe profiles (**a**, **b**, **c**, *BRAF* – red; 7p control – green; **d**, centromeric *BRAF* – green; telomeric *BRAF* – red) indicate normal *BRAF* in GG02 **(a)** and a classic ‘doublet’ pattern with these probes for duplicated *BRAF* in GG21 (**b**, arrows). In GG17 **(c, d)**, FISH preparations indicated a complex alteration; probe profiles showed both duplication of *BRAF* and a monallelic separation of duplicated *BRAF*.

iFISH demonstrated *BRAF* duplication in 9 of 11 (82%) group II tumors (Figure 3), but in none of the group I tumors (Table 1). One group II tumor, GG17, demonstrated *BRAF* duplication and a potential *BRAF* fusion, the latter on the basis of a ‘break-apart’ probe profile that showed one (normal) overlapping pair of signals and one ‘split’ pair of signals (Figure 3d). *KIAA1549-BRAF* fusions were found in all 9 group II tumors with *BRAF* duplication, but in no other group I or group II tumor. Three *KIAA1549-BRAF* fusion variants were identified; exon16:exon9, exon15:exon9, and exon16:exon11 (Table 1).

BRAF:p.V600E mutations were detected in 7 of 16 (43%) group I tumors, but in no group II tumor. No mutations at KRAS:p.G12 or KRAS:p.Q61 were identified across the tumor cohort. No tumors showed evidence of an *ETV6-NTRK3* fusion.

### Radiological features

Of 27 patients in the study cohort, MRI with and without contrast and diffusion-weighted imaging at presentation were available for review in 25 and 16, respectively. Of imaged group I tumors, 3/14 were well-circumscribed, compared to 7/11 in group II (Table 1). Among group I tumors, the most common primary site of tumor involvement was the medulla, followed by the middle cerebellar peduncle (MCP), with secondary involvement of the pons (8/14), MCP (5/14), cervical spinal cord (4/14), cerebellar hemisphere (3/14) and vermis (1/14). Among group II tumors, the vermis and spinal cord were the most frequent sites of primary involvement. One group II tumor was centered in the medulla with secondary involvement of the MCP, pons and cervical cord, and another was centered on the midbrain with secondary thalamic involvement. Three of five vermian tumors had secondary involvement of the cerebellar hemispheres. All imaged tumors enhanced, but group II tumors were more frequently cystic or necrotic and hemorrhagic; no group I tumor demonstrated hemorrhage on MRI. A triad of radiological features, encompassing hemorrhage, midline location, and the presence of cysts or necrosis, was able to separate group I and group II tumors with a sensitivity of 91% and specificity of 100%.

## Discussion

Gangliogliomas are rare low-grade neuroepithelial tumors of the CNS consisting of admixed mature glial and neuronal elements [2,4]. Most arise in the temporal lobe or other supratentorial sites, but they occasionally occur in the posterior fossa or spinal cord [2,5,6,20]. Most classic gangliogliomas contain an idiosyncratic glial component that combines pilocytic and fibrillary phenotypes, and in a significant proportion of tumors this element infiltrates surrounding parenchyma blurring the border between tumor and normal tissue.

On the basis of our clinical experience with a few infratentorial low-grade glioneuronal tumors that were largely pilocytic astrocytomas but exhibited foci of gangliocytic differentiation, this study tested the hypothesis that gangliogliomas of the posterior fossa and spinal cord can be divided into distinct morphological groups and that these groups would also be characterized by distinct molecular alterations. In a series of 27 gangliogliomas, we found that 16 (59%) had the features of a classic ganglioglioma with admixed neuronal and glial elements, while 11 (41%) would have been classified as pilocytic astrocytomas, were it not for the presence of a few circumscribed collections of cells with gangliocytic differentiation. Our detailed review of patients’ neuroimaging indicated that the two groups of tumors could also be differentiated by specific radiological characteristics; a triad of features was able to separate the two morphologic groups with 91% sensitivity and 100% specificity. The detailed pathology of a large series of infratentorial gangliogliomas has not been previously reported, but one study noted that a cerebellar ganglioglioma demonstrated a prominent pilocytic component [11].

Recent genomic studies have defined the genetic alterations of most low-grade neuroepithelial tumors. Alterations in genes involved in the MAPK pathway dominate; *KIAA1549-BRAF* fusions characterize PAs, occurring in approximately 90% of posterior fossa tumors but at lower frequencies in spinal cord and supratentorial tumors [12,17-19]. Some PAs demonstrate an alternative *BRAF* rearrangement, where *BRAF* partners with another gene, including *FAM131B, MACF1, FXR1, RNF130, CLCN6, MKRN1* and *GNAI1*[17,19,21]. BRAF:p.V600E mutations occur in PXAs (~70%), gangliogliomas (~25%), and WHO grade II diffuse astrocytomas (~20%) [11,14,19,22,23]. Rarely, mutations of *KRAS* are found in a PA or grade II diffuse glioma [12,19,24], and an *ETV6-NTRK3* fusion gene has been reported in a PXA [19]. However, such genetic abnormalities were not harbored by those gangliogliomas in which we were unable to show a *KIAA1549-BRAF* fusion or BRAF:p.V600E mutation. Low-grade neuroepithelial tumors presenting in childhood rarely contain an IDH1:p.R132H mutation. This mutation is regarded as a hallmark of adult-type disease, but can occur in adolescents with a WHO grade II diffuse glioma [19]. Another rare glioneuronal tumor of the posterior fossa, the rosette-forming glioneuronal tumor of the fourth ventricle, has a distinct morphology from the two types of ganglioglioma in our study [25,26]. Additionally, it is not characterized by *KIAA1549-BRAF* fusion or BRAF:p.V600E mutation [27].

Our analysis of molecular alterations in infratentorial gangliogliomas has revealed a clear distinction between two morphological groups. Seven of sixteen (44%) tumors in group I, with features of a classic ganglioglioma, harbored a BRAF:p.V600E mutation. This mutation is the most common genetic alteration yet found in gangliogliomas and links this infratentorial morphologic group to typical cerebral gangliogliomas. Group II contained tumors that were largely pilocytic astrocytomas, but with foci of gangliocytic differentiation; 82% of these tumors were characterized by a *KIAA1549-BRAF* fusion gene, which is the hallmark of pilocytic astrocytomas. Therefore, the frequency of *KIAA1549-BRAF* fusions in infratentorial PAs and gangliogliomas appears very similar.

## Conclusions

We have provided clear evidence of the separation of posterior fossa and spinal gangliogliomas into two groups distinguished by their morphological, radiological and genetic characteristics. One group should be regarded as classic gangliogliomas, while on the basis of molecular data the other might be better classified as pilocytic astrocytomas with gangliocytic differentiation.

## Competing interests

The authors declare that they have no competing interests.

## Authors’ contributions

Pathology review: KG, TR, DWE. Molecular analysis: KG, WO, JDD, CP, RC-U. Clinical data collection: IQ, RGT. Radiology review: JHH. Manuscript writing: KG, RGT, DWE. Study conception and oversight: DWE. All authors read and approved the final manuscript.
